# Identifying Critical Non-Catalytic Residues that Modulate Protein Kinase A Activity

**DOI:** 10.1371/journal.pone.0004746

**Published:** 2009-03-09

**Authors:** Eileen J. Kennedy, Jie Yang, Lorraine Pillus, Susan S. Taylor, Gourisankar Ghosh

**Affiliations:** 1 Department of Chemistry and Biochemistry, University of California San Diego, La Jolla, California, United States of America; 2 Division of Biological Sciences, Section of Molecular Biology and UCSD Moores Cancer Center, University of California San Diego, La Jolla, California, United States of America; 3 Howard Hughes Medical Institute, University of California San Diego, La Jolla, California, United States of America; Temasek Life Sciences Laboratory, Singapore

## Abstract

**Background:**

Distal interactions between discrete elements of an enzyme are critical for communication and ultimately for regulation. However, identifying the components of such interactions has remained elusive due to the delicate nature of these contacts. Protein kinases are a prime example of an enzyme with multiple regulatory sites that are spatially separate, yet communicate extensively for tight regulation of activity. Kinase misregulation has been directly linked to a variety of cancers, underscoring the necessity for understanding intramolecular kinase regulation.

**Methodology/Principal Findings:**

A genetic screen was developed to identify suppressor mutations that restored catalytic activity *in vivo* from two kinase-dead Protein Kinase A mutants in *S. cerevisiae*. The residues defined by the suppressors provide new insights into kinase regulation. Many of the acquired mutations were distal to the nucleotide binding pocket, highlighting the relationship of spatially dispersed residues in regulation.

**Conclusions/Significance:**

The suppressor residues provide new insights into kinase regulation, including allosteric effects on catalytic elements and altered protein-protein interactions. The suppressor mutations identified in this study also share overlap with mutations identified from an identical screen in the yeast PKA homolog Tpk2, demonstrating functional conservation for some residues. Some mutations were independently isolated several times at the same sites. These sites are in agreement with sites previously identified from multiple cancer data sets as areas where acquired somatic mutations led to cancer progression and drug resistance. This method provides a valuable tool for identifying residues involved in kinase activity and for studying kinase misregulation in disease states.

## Introduction

Protein kinases comprise one of the largest gene families and are responsible for regulating cellular processes throughout all stages of growth and development, including gene transcription [1], metabolism [2], cell cycle regulation [3] and apoptosis [4]. Critical regulatory roles performed by kinases are underscored by the occurrence of numerous cancer phenotypes when kinase activity is altered or misregulated [5].

**Figure 1:**
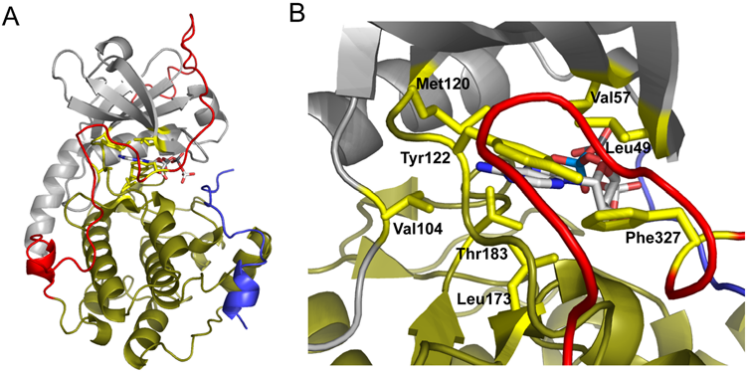
Residues highlighted to interact with the nucleotide base in PKA. When PKA is in an ATP-bound state, the small lobe (gray) and glycine-rich loop move toward the large lobe (sand) to enclose the nucleotide-binding pocket around ATP. *A*, A global view of PKA in a nucleotide-bound state. Eight residues have been identified to interact with the nucleotide base: Leu 49, Val 57, Val 104, Met 120, Tyr 122, Leu 173, Thr 183, and Phe 327 (highlighted yellow). The bound nucleotide is represented as a ball-and-stick figure. The C-terminal tail is colored red, and PKI is colored magenta. *B*, Zoomed view into the nucleotide binding pocket. The nucleotide binding pocket is largely preformed with the exception of Val 57, Met 120, Leu 173, and Phe 327 which undergo structural changes upon nucleotide binding. Structure adapted from PDB accession number 1ATP and modeled using Pymol.

The kinase superfamily shares a conserved core that contains several invariant amino acids that contribute to activity [6]. Of the kinase superfamily, Protein Kinase A (PKA) is perhaps the best characterized. The tetrameric holoenzyme of PKA is composed of two catalytic subunits that possess kinase activity, and two regulatory subunits that are responsive to cAMP [7], [8]. The kinase domain is composed of a small lobe and a large lobe. The active site cleft resides between the lobes and is lined with multiple conserved residues that directly interact with the nucleotide upon binding (Figure 1). The ATP binding pocket is largely preformed but undergoes small structural rearrangements during nucleotide binding, whereas the active site cleft is dynamic and undergoes large structural changes to allow for nucleotide binding and catalysis [9]. In order to achieve tightly regulated catalytic activity, distal regions of the kinase must allosterically communicate with one another in order to synchronize structural changes [10]–[12].

Identifying distal components involved in kinase activity has remained elusive as a consequence of small structural changes and weak residue interactions. In order to expose these modulators throughout conserved and non-conserved elements of a kinase, a structurally non-biased approach was utilized. A genetic screen was developed to identify suppressor mutations that could restore catalytic activity from a kinase-dead mutant of PKA. Mutations were introduced to generate suppressor libraries, and libraries were screened in an *S. cerevisiae* strain devoid of native PKA expression. Since PKA activity is essential for survival, a viability screen was used to identify mutations that could suppress a catalytically inactive mutant. Screening revealed previously unidentified residues that are critical for PKA activity. In this report, we identify suppressor mutations that were isolated from two discrete genetic backgrounds. The mutations identified in this study were previously unknown modulators of kinase activity and point to multiple modes for achieving suppression. This genetic screen may be applied as a tool to investigate the relationship between kinase disregulation and various disease states.

## Results

### The *prkaca-L173A* and *prkaca-F327A* alleles cannot support essential PKA function

**Figure 2:**
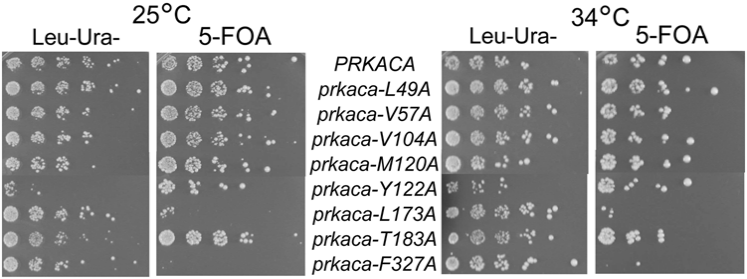
*prkaca-L173A* and *prkaca-F327A* cannot support viability in a *tpk* null strain. Point mutations were introduced at the nucleotide binding sites indicated in Figure 1*B*. Coexpression of wild-type and mutant alleles of PKA is demonstrated on Leu-Ura- selection plates. Expression of the mutants alone is shown on 5-FOA plates. All mutants tested, except *prkaca-L173A* and *prkaca-F327A*, were viable at permissive and elevated temperatures.

As a basis for the genetic screen, we sought to identify nucleotide binding pocket mutations that would render the kinase catalytically inactive *in vivo*. Several key residues within the nucleotide binding pocket that interact with the nucleotide base were mutated in the murine isoform of PKA (L49A, V57A, L104A, M120A, Y122A, L173A, T183A and F327A) [9]. The mutants were characterized *in vivo* using the model organism *S. cerevisiae* as it affords a genetic background that can be readily manipulated. Since PKA activity is essential for survival in yeast [13], a synthetically lethal strain was created by deleting all three genes encoding PKA (*TPK1*, *TPK2*, and *TPK3*). Plasmid shuffling revealed that viability could be fully maintained in this strain at both permissive and elevated temperatures by expressing the murine isoform of PKA (Figure 2) as previously demonstrated [14]. In order to identify single mutations that could render PKA inactive, each site was individually mutated and transformed in the *tpk* null strain to evaluate kinase function. The *tpk* null strain was initially maintained by expression of wild-type *TPK1* from a *URA3*-marked plasmid since it has the highest degree of sequence identity to the murine PKA homolog. Wild-type and mutant PKA proteins were expressed using the endogenous *TPK1* promoter in *LEU2*-marked plasmids. Each PKA mutant was subsequently tested using standard “plasmid shuffle” assays to determine whether essential PKA function was present in the absence of *TPK1* expression [15].

**Figure 3:**
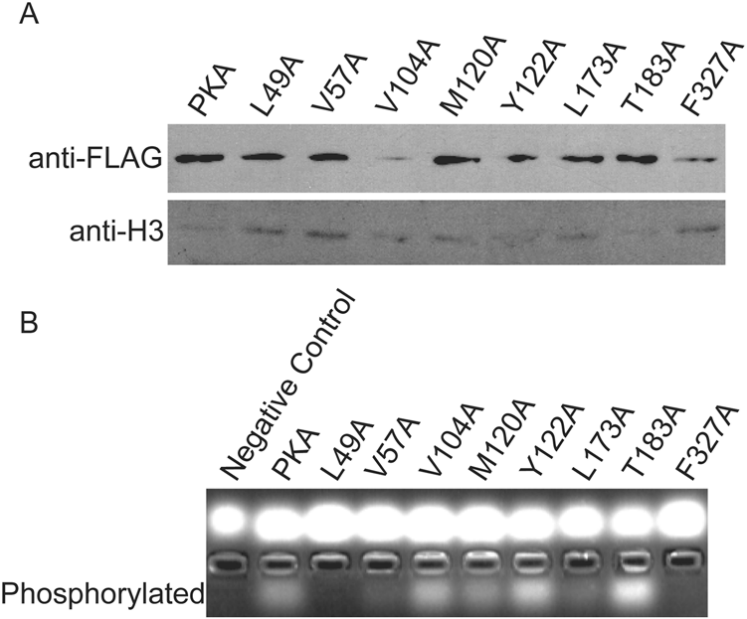
The L173A mutant protein is stably expressed but inactive, whereas the F327A mutant has lowered protein expression. *A*, Protein expression of each PKA mutant were measured. Of the two mutants that could not complement synthetic lethality in the *tpk* null strain, F327A also demonstrated very low protein expression. *B*, The catalytic activity of each PKA mutant was determined. Both L173A and F327A mutants had nearly undetectable catalytic activity.

Growth resulting from co-expression of wild-type and mutant plasmids (Leu- Ura-) was compared to that of the mutant plasmid alone (5-fluoroorotic acid, 5-FOA) (Figure 2). When co-expressed with wild-type Tpk1, most PKA mutants grew comparably to the wild-type at both permissive (25°C) and elevated temperatures (34°C). However, a significant growth defect was observed for two mutants: *prkaca-L173A* and *prkaca-F327A*. At both temperatures, the *prkaca-F327A* mutant was completely non-viable and had very low steady-state protein expression (Figure 3A). The *prkaca-L173A* mutant demonstrated very weak growth only at the highest concentration. Both mutants demonstrated significantly reduced catalytic activity *in vitro* (Figure 3B). Previous crystallographic analysis reveals that of the eight residues tested, four residues retain their structural positions (L49, L104, Y122 and T183) whereas the other four (V57, M120, L173, and F327) undergo structural changes during nucleotide binding [16]. The dramatic inability of the *prkaca-L173A* and *prkaca-F327A* mutants to maintain viability in the *tpk* null strain may indicate that residues that undergo structural rearrangement may play a more significant role in regulating nucleotide binding for PKA activity.

### Intragenic suppression restores catalytic activity to *prkaca* mutants

Using the nonviable *prkaca* site-directed mutants identified above, a genetic approach was employed to identify suppressor mutations that could overcome the non-viable phenotype. The *prkaca-L173A* and *prkaca-F327A* mutants were both selected for suppressor screening since they provide two unique backgrounds: L173A lies within the conserved kinase core while F327A lies within the non-conserved C-terminal tail. Plasmid libraries were constructed after random mutagenesis of the *prkaca-L173A* and *prkaca-F327A* mutant alleles. These were transformed and screened for restoration of viability to the *tpk* null strain using standard plasmid shuffling. Mutation rates of libraries varied from approximately 1–20 mutations per 1000 base pairs. Approximately 25,000 transformants were screened for each allele, and suppressors were sequenced to identify the acquired mutations. The suppressors were isolated from each background separately.

Suppressor mutations were screened based upon their ability to restore viability to the *tpk* null strain and were more readily identified from the *prkaca-F327A* background than from the *prkaca-L173A* background (Tables 1 and 2). Additionally, suppressors from the *pkraca-L173A* background required at least two additional amino acid changes in order to suppress the L173A mutation. Due to the variance noted between the two discrete genetic backgrounds during screening, it appears that the modes of suppression are dependent upon the effects caused by the initial mutation.

**Table 1:** Suppressor mutations identified from the *prkaca-L173A* background.

Suppressor	Changes in Suppressors
Suppressor 27	T153S, L160N, S212N
Suppressor 28	T153S, L160N, S212N
Suppressor 31	Q181K, T278S
Suppressor 51	M58V, R190C
Suppressor 52	M58V, R190C
Suppressor 53	M58V, R190C
Suppressor 54	M58V, R190C
Suppressor 55	Wild-type reversion
Suppressor 57	M58V, R190C
Suppressor 58	K28N, I305L, E311V, I315L
Suppressor 59	K28N, I305L, E311V
Suppressor 60	K28N, E311V
Suppressor 61	K21Q, K28N, R308K, E311V
Suppressor 62	Wild-type reversion
Suppressor 63	R190C, R194H, K292Q, I303V

**Table 2:** Suppressor mutations identified from the *prkaca-F327A* background.

Suppressor	Changes in Suppressors
Suppressor 1	Y229S
Suppressor 3	G186C
Suppressor 4	T51P
Suppressor 5	F327S, L173V
Suppressor 7	E17R
Suppressor 12	M120T, L40S
Suppressor 14	K285P
Suppressor 32	V310A
Suppressor 38	H62L, Q149H
Suppressor 39	T37K, D75Q, Q149H, K189N, R190H, V226A, Q242H
Suppressor 40	Q35L, A38T, E140Y, Q149H, E170D, G178V, I180L, R190H, C199S, G214K
Suppressor 41	K28E, L40S, R93S
Suppressor 42	Q35L, K295E, I305L
Suppressor 43	L40S, K189T, T299P, Q307H, F318I
Suppressor 44	L40S, K47T, Y164H
Suppressor 46	L40S, K63N, L95M
Suppressor 47	M71I, V251I, F257I
Suppressor 48	L40S, R308K, I315L
Suppressor 49	V251F
Suppressor 66	F108I, Y215C
Suppressor 67	L40S, L157M
Suppressor 68	L40P
Suppressor 69	L40S
Suppressor 70	T37I, L40P, G282A

### Suppression of the *prkaca-L173A* mutant

**Figure 4:**
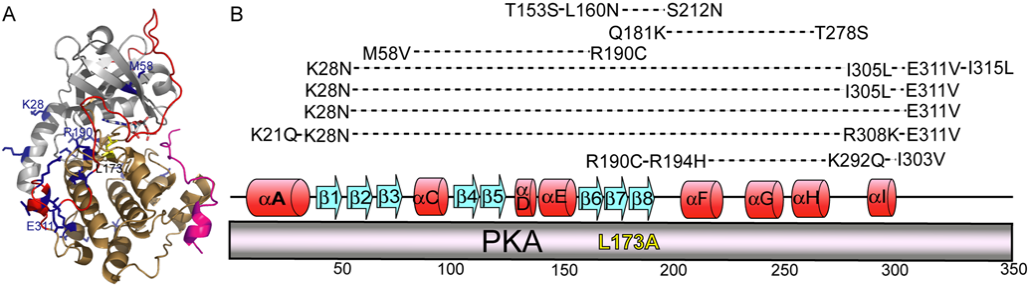
Suppressors identified from the *prkaca-L173A* background. *A*, The original L173A mutation is highlighted yellow and the suppressor mutations are shown in blue. PKA features are highlighted as follows: small lobe, gray; large lobe, sand; C-terminal tail,red; PKI: magenta. Some frequently isolated suppressor mutations are labeled. Mutations are shown using PDB file 1ATP and modeled using PyMol. *B*, Multiple mutations isolated from the same suppressor are connected by a dashed line and generally aligned with secondary structural elements of PKA. Secondary structural elements are represented where alpha-helices are red and beta-sheets are blue.

The *prkaca-L173A* suppressors were first investigated (Figure 4). The original L173A mutation is buried within the nucleotide binding pocket of PKA and directly interacts with the nucleotide base. Notably, some suppressors were independently isolated several times. Of these, the most commonly isolated suppressor was one that had acquired mutations at sites M58V and R190C. Both residue sites are conserved among the PKA family. Met58 is positioned within the β-sheets of the small lobe and is partially buried between the small lobe and the C-terminal tail in the nucleotide-bound state. Arg190 is also partially buried and points away from the nucleotide binding pocket near the hinge region flanking the magnesium-positioning DFG loop. Both residues are members of an intramolecular interaction network that provides crosstalk between the hydrophobic lining and the gly-rich and DFG loops, respectively, of AGC kinases [10]. The high frequency of isolating this suppressor pair may be due to the simultaneous effects on multiple known regulatory components of PKA.

Another commonly isolated suppressor from the *prkaca-L173A* background acquired a pair of mutations: K28N and E311V. Interestingly, both residues reside outside of the conserved kinase core. Lys28 is a solvent-exposed residue in the non-conserved A-helix of the small lobe. The A-helix regulates kinase activity by acting as a communication bridge between the small and large lobes, and also partially anchors the C-helix for nucleotide positioning. Glu311 is positioned in the C-terminal tail and acts to help anchor the tail to the large lobe as it undergoes structural rearrangement upon nucleotide binding to act as a regulator for access to the active site. Kinase activity may have been restored to the *prkaca-L173A* background by coupling effects stemming from non-conserved elements to alter the activity of the conserved kinase core.

### Suppression of the *prkaca-F327A* mutant

**Figure 5:**
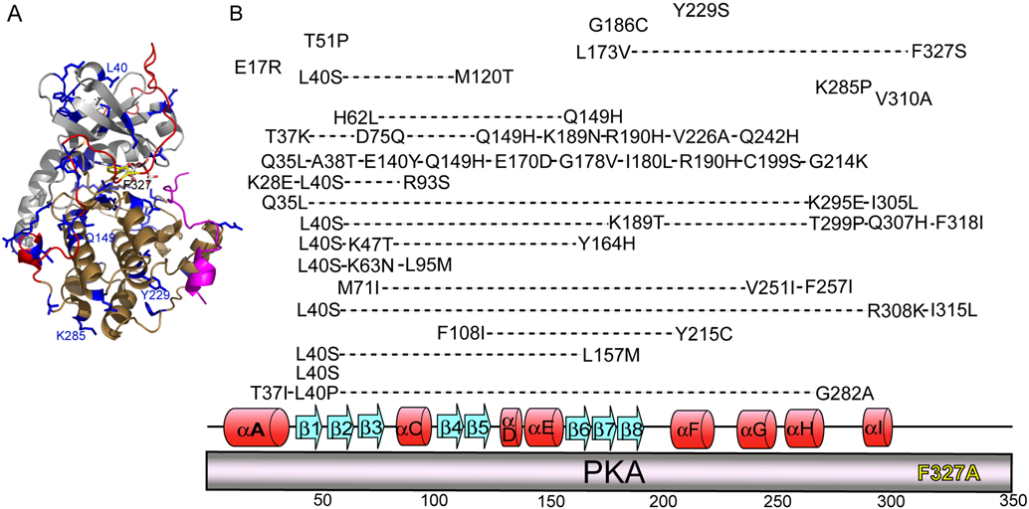
Suppressor mutations from the *prkaca-F327A* background. *A*, Highlighted suppressor mutations identified from the *prkaca-F327A* background. The original F327A mutation is highlighted in yellow, and suppressor mutations are shown in blue. PKA features are highlighted as follows: small lobe, gray; large lobe, sand; C-terminal tail,red; PKI: magenta. Some frequently highlighted mutations are labeled. The image was made using PDB file 1ATP and modeled using PyMol. *B*, Mutations isolated from the same suppressor are connected by a dashed line. Single suppressor mutations are listed above the corresponding secondary structural elements of PKA. Secondary structural elements are represented where alpha-helices are red and beta-sheets are blue.

In order to determine how suppression could be achieved from a key catalytic residue outside of the conserved kinase core, suppression of the *prkaca-F327A* mutant was subsequently examined. The *prkaca-F327A* background demonstrates the significance of a non-conserved element on kinase activity. The F327A mutation may prevent the C-terminal tail from fully undergoing the structural transition after a nucleotide is bound, deeming it both inactive and structurally unstable (Figure 3). Viable suppressors were isolated from the *prkaca-F327A* background (Table 2 and Figure 5). Of note, suppressors were isolated at a much higher rate as compared to the *prkaca-L173A* background and could be isolated from libraries with low mutation rates.

As seen in the *prkaca-L173A* background, some suppressors were identified multiple times. The most frequently isolated mutation occurred at Leu40. Leu40 is part of the conserved kinase core and is the first residue of β-strand 1 in the small lobe. This conserved residue also belongs to an interaction network connecting the hydrophobic lining of AGC kinases with the glycine-rich loop [10]. The acquired L40S/P mutation likely augments propagated signaling from the hydrophobic lining of the catalytic domain to the glycine-rich loop.

Another frequently isolated suppressor was Q149H. This site is located on the E-helix of the large lobe and interacts with the proximal end of the C-terminal tail. In a nucleotide-bound state, Gln149 becomes completely buried by the C-terminal tail. The Q149H suppressor may promote packing interactions within the large lobe to support anchoring of the C-terminal tail as it undergoes the structural transition during nucleotide binding. Additionally, Q149 is involved in allosteric regulation of the DFG loop during transitions between active and inactive states [12]. The compensatory Q149H mutation may ease the allosteric regulation to allow for suppression of the original F327A mutation.

## Discussion

Many of the acquired suppressor mutations would not have necessarily been predicted to significantly impact kinase activity. As such, it was not obvious in many cases how suppression was achieved. Of note, many suppressor mutations are structurally distal to the original L173A mutation. All of the *prkaca-L173A* suppressors acquired at least two additional mutations, perhaps indicating that overcoming a momentous deficiency stemming from loss of a key catalytic residue within the kinase core required multiple changes throughout the kinase.

In contrast to suppressors identified from the *prkaca-L173A* background, it is interesting to note that many of the acquired mutations from the *prkaca-F327A* background are at sites that are solvent-exposed. It is possible that these mutations affect substrate or other protein-protein interactions, leading to enhanced or altered affinities for inhibitor interactions or protein complex formation to ultimately suppress the original prkaca-F327A mutation. Additionally, multiple *prkaca-F327A* suppressors acquired a proline mutation. Proline inherently decreases localized mobility, thereby inhibiting conformational malleability. In this instance, buried Pro mutations may disallow conformational changes to impact allosteric regulation for catalysis, but surface Pro mutations may alter interactions involving substrate binding or protein-protein interactions.

A noteworthy example of a proline suppressor mutation was identified in the F327A/K285P suppressor. Further structural and biochemical characterization was performed on this suppressor to elucidate the mechanism of suppression [17]. It was found that F327 and the dynamic part of the C-tail flanking this site play an essential role in regulating kinase activity by participating in ATP binding, coordinating the positioning of the Glycine-rich Loop for catalysis and by stabilizing the linker joining the two lobes of the kinase core. Thus, the F327A mutation caused a loss of catalytic function by distorting the abovementioned elements. However, biochemical studies revealed that the double mutant remained catalytically defective, yet kinase function was restored *in vivo*. Suppression was achieved by reducing the inhibitory interactions with either the RII-subunits or the yeast homolog Bcy1p. Even though the mutations are distal to one another, crystallographic studies revealed that K285 provides a key contact point for interactions with the regulatory subunits. The side chain of Lys285, located in the αH-αI Loop of the large lobe, is typically solvent exposed and often disordered in crystal structures where the C-subunit is not bound to a regulatory subunit. Therefore, its functional importance was not previously appreciated. The suppressor mutation prevented the regulatory subunit from binding to the kinase domain, thereby allowing the kinase to remain constitutively active. Further, the single mutants K285P or F327A had a modest effect on inhibition by RIIα, however, a synergistic negative effect was observed for the double mutant. This suggests an allosteric connection of the αH-αI Loop to the active site. Detailed analysis of this suppressor provides an example of how distal sites of PKA are functionally integrated in order to regulate PKA activity.

### Functional differences between PKA and Tpk2

In this study, several key residues within the nucleotide-binding site of PKA were tested *in vivo* for their necessity in kinase function in an *S. cerevisiae* strain devoid of native PKA expression. Despite site conservation among the kinase superfamily, only two sites, L173 and F327, demonstrated a loss of viability when mutated to Ala. The biological consequences of these mutations were previously tested *in vivo* using the PKA homolog Tpk2 [18]. Interestingly, although viability was lost in the *tpk2-T183V* mutant, absolutely no effects were seen for the same mutant in PKA. Likewise, *prkaca-L173A* and *prkaca-F327A* caused loss of viability in the *tpk* null strain, but no consequences were evident for the corresponding Tpk2 mutants. This demonstrates that although the nucleotide binding pocket is conserved among the kinase superfamily, their functional roles may not be conserved.

Some identical suppressor mutations were identified from screening against both of the *prkaca* backgrounds as well as the yeast homolog *tpk2-T183V* [18]. One example of this is the acquisition of mutations at site R190. This residue lies within the conserved kinase core and forms a socket with R93 at the end of the C-helix. This socket interacts with W30 and F26 of the A-helix to anchor and position the C-helix for catalytic activity. This socket also provides support to the A-helix to maintain conformational rigidity between the large and small lobes. Identification of acquired mutations at R190 from two distinct genetic backgrounds of PKA as well as the yeast homolog Tpk2 underscores the significance of the interactions between the A- and C-helices on kinase function.

There is a clear link between cancer progression and a variety of conserved, somatic mutations that accumulate over time in kinases. A recent study analyzing multiple cancer data sets has identified four residues within the catalytic core of multiple kinases that play a dominant role in tumor progression [19]. In PKA, these positions are L49, R190, M120 and G126. Our genetic screening against three unique backgrounds using two PKA homologs yielded acquisition of suppressor mutations at positions R190 and M120 for several suppressors. This not only validates the genetic screen as a method for identifying residues that modulate catalytic activity, but also reconfirms the significance of these residues in drug resistance and cancer.

### Genetic screening as a tool for studying kinase regulation

Although crystal structures have shed tremendous light on catalytic components of kinases, the more subtle regulatory interactions distributed throughout the kinase have been difficult to distinguish. These results suggest that viability-based genetic screening can identify suppressors that indirectly reverse the functional defectiveness derived from the original mutation by compensatory effects distributed throughout the kinase. Due to the significance of kinases in a variety of diseases, understanding kinase regulation is critical. This method provides a platform for *in vivo* identification of previously unsuspected sites that significantly impact PKA regulation. The genetic screen presented in this study may act as a tool to broaden our understanding of kinase regulation and can be applied to study kinase misregulation in disease states.

## Materials and Methods

### Strains and Media

The yeast strain used in this study was derived from the *Saccharomyces* Genome Deletion Project [20]. The strain, LPY 06292 has the following genotype: *MATα his3-1 leu2-0 lys2-0 met15-0 ura3-0 tpk1Δ::KANMX tpk2Δ::KANMX tpk3Δ::KANMX*. To survive, it carries a *CEN*, *URA3*-marked plasmid bearing wild-type *TPK1* (pLP2024). Where indicated, wild-type and *PKA* mutants were co-expressed in a *CEN*, *LEU2*-marked vector [21]. Growth was at 30°C unless otherwise specified and standard techniques for yeast manipulation were performed [15]. Cultures were grown in either yeast extract/ peptone/ dextrose medium (YPD), Leu- Ura- drop-out medium, or Leu- 5-FOA medium as indicated that was prepared as described [22].

### Generation of PKA pocket mutations and screening for restored PKA function

Point mutations were introduced into sequences encoding the nucleotide-binding pocket of *Mus musculus* PKA-Cα (Gene symbol: Prkaca) using the QuikChange Site-Directed Mutagenesis Kit (Stratagene, La Jolla, CA) using the following forward primers and their reverse complements, respectively:

Leu49Ala:
5′-AGAATCAAGACCGCGGGCACCGGATCC,
5′-GGATCCGGTGCCCGCGGTCTTGAATTCT;
Val57Ala:
5′-TCCTTTGGCCGAGCGATGCTGGTGAAG,
5′-CTTCACCAGCATCGCTCGGCCAAAGGA;
Val104Ala:
5′-TTCCCGTTCCTGGCCAAACTTGAATTC,
5′-GAATTCAAGTTTGGCCAGGAACGGGAA;
Met120Ala:
5′-CTGTACATGGTGGCCGAGTATGTAGCT,
5′-AGCTATATACTCGGCCACCATGTACAG;
Tyr122Ala:
5′-ATGGTCATCGAGGCTGTGGCCGGTGGC,
5′-GCCACCGGCCACAGCCTCCATGACCAT;
Leu173Ala:
5′-CCCGAGAATCTTGCGATCGACCAGCAG,
5′-CTGCTGGTCGATCGCAAGATTCTCGGG;
Thr183Ala:
5′-TATATTCAGGTGGCCGACTTCGGTTTT,
5′-AAAACCGAAGTCGGCCACCTGAATATA;
Phe327Ala:
5′-GACACTAGTAACGCTGACGACTATGAG,
5′-CTCATAGTCGTCAGCGTTACTAGTGTC

Intragenic suppressor libraries of either the *prkaca-L173* or *prkaca-F327A* mutants were generated as previously described [18]. Coding sequence templates of *prkaca* bearing the mutations encoding either L173A or F327A were used as target templates for PCR amplification in pRS315 plasmids bearing 2000 bp upstream and 1000 bp downstream flanking sequences from the closest PKA-Cα homolog in *S. cerevisiae*, *TPK1*.

### Phenotypic analysis of strains with point mutations in PKA

Cultures were grown in Leu- Ura- medium. Cell densities were normalized to OD_600_ = 1.0 and were 5-fold serially diluted in sterilized water. Dilutions were plated onto selective medium and grown at the indicated temperatures for 6 days.

### Protein expression, immunodetection, purification of FLAG-labeled PKA catalytic subunit, and catalytic kinase activity assays

Immunodetection, protein purification, and catalytic activity assays were performed as previously described [18].

## References

[1] Manning G, Whyte DB, Martinez R, Hunter T, Sudarsanam S (2002). The protein kinase complement of the human genome.. Science.

[2] Walsh DA, Perkins JP, Krebs EG (1968). An adenosine 3′,5′-monophosphate-dependant protein kinase from rabbit skeletal muscle.. J Biol Chem.

[3] Hirai H, Kawanishi N, Iwasawa Y (2005). Recent advances in the development of selective small molecule inhibitors for cyclin-dependent kinases.. Curr Top Med Chem.

[4] Heyninck K, Beyaert R (2005). A novel link between Lck, Bak expression and chemosensitivity.. Oncogene.

[5] Lengyel E, Sawada K, Salgia R (2007). Tyrosine kinase mutations in human cancer.. Curr Mol Med.

[6] Hanks SK, Hunter T (1995). Protein kinases 6. The eukaryotic protein kinase superfamily: kinase (catalytic) domain structure and classification.. Faseb J.

[7] Knighton DR, Zheng JH, Ten Eyck LF, Xuong NH, Taylor SS (1991). Structure of a peptide inhibitor bound to the catalytic subunit of cyclic adenosine monophosphate-dependent protein kinase.. Science.

[8] Kim C, Xuong NH, Taylor SS (2005). Crystal structure of a complex between the catalytic and regulatory (RIalpha) subunits of PKA.. Science.

[9] Taylor SS, Yang J, Wu J, Haste NM, Radzio-Andzelm E (2004). PKA: a portrait of protein kinase dynamics.. Biochim Biophys Acta.

[10] Masterson LR, Mascioni A, Traaseth NJ, Taylor SS, Veglia G (2008). Allosteric cooperativity in protein kinase A.. Proc Natl Acad Sci U S A.

[11] Yang J, Ten Eyck LF, Xuong NH, Taylor SS (2004). Crystal structure of a cAMP-dependent protein kinase mutant at 1.26A: new insights into the catalytic mechanism.. J Mol Biol.

[12] Ten Eyck LF, Taylor SS, Kornev AP (2008). Conserved spatial patterns across the protein kinase family.. Biochim Biophys Acta.

[13] Toda T, Cameron S, Sass P, Zoller M, Wigler M (1987). Three different genes in S. cerevisiae encode the catalytic subunits of the cAMP-dependent protein kinase.. Cell.

[14] Zoller MJ, Yonemoto W, Taylor SS, Johnson KE (1991). Mammalian cAMP-dependent protein kinase functionally replaces its homolog in yeast.. Gene.

[15] Amberg DC, Burke DJ, Strathern JN (2005).

[16] Akamine P, Madhusudan, Wu J, Xuong NH, Ten Eyck LF (2003). Dynamic features of cAMP-dependent protein kinase revealed by apoenzyme crystal structure.. J Mol Biol.

[17] Yang J, Kennedy EJ, Wu J, Deal MS, Brown S (2009). Contribution of Non-Catalytic Core Residues to Activity and Regulation in Protein Kinase A.. J Biol Chem.

[18] Kennedy EJ, Pillus L, Ghosh G (2008). Identification of functionally distinct regions that mediate biological activity of the Protein Kinase A homolog Tpk2.. J Biol Chem.

[19] Torkamani A, Schork NJ (2008). Prediction of cancer driver mutations in protein kinases.. Cancer Res.

[20] Winzeler EA, Shoemaker DD, Astromoff A, Liang H, Anderson K (1999). Functional characterization of the S. cerevisiae genome by gene deletion and parallel analysis.. Science.

[21] Sikorski RS, Michaud WA, Tugendreich S, Hieter P (1995). Allele shuffling: conjugational transfer, plasmid shuffling and suppressor analysis in Saccharomyces cerevisiae.. Gene.

[22] Sherman F (2002). Getting started with yeast.. Methods in Enzymology.

